# Recent Developments in Solid-Phase Extraction for Near and Attenuated Total Reflection Infrared Spectroscopic Analysis

**DOI:** 10.3390/molecules21050633

**Published:** 2016-05-14

**Authors:** Christian W. Huck

**Affiliations:** Institute of Analytical Chemistry and Radiochemistry, CCB-Center for Chemistry and Biomedicine, Leopold-Franzens University, Innrain 80/82, 6020 Innsbruck, Austria; Christian.W.Huck@uibk.ac.at; Tel.: +43-512-507-57304; Fax: +43-512-507-57399

**Keywords:** solid-phase extraction, near-infrared spectroscopy, mid-infrared spectroscopy, attenuated total reflection, enrichment, selectivity, sensitivity, efficiency

## Abstract

A review with more than 100 references on the principles and recent developments in the solid-phase extraction (SPE) prior and for *in situ* near and attenuated total reflection (ATR) infrared spectroscopic analysis is presented. New materials, chromatographic modalities, experimental setups and configurations are described. Their advantages for fast sample preparation for distinct classes of compounds containing different functional groups in order to enhance selectivity and sensitivity are discussed and compared. This is the first review highlighting both the fundamentals of SPE, near and ATR spectroscopy with a view to real sample applicability and routine analysis. Most of real sample analyses examples are found in environmental research, followed by food- and bioanalysis. In this contribution a comprehensive overview of the most potent SPE-NIR and SPE-ATR approaches is summarized and provided.

## 1. Introduction

The development of a complete analytical strategic method requires the inclusion of a number of steps, starting from sample collection and ending with the final result report. Intermediate steps include sample storage, preparation, isolation, identification and quantification of analytes. Thereby, sample preparation represents a tedious and time-consuming step, which is also very often a source of imprecision and inaccuracy of the overall analysis. The basic principal objective of sample preparation for chromatographic as well as infrared spectroscopic analysis are dissolution of analytes in a suitable solvent and removal from the solution of as many interfering compounds as possible. For this purpose, solid-phase extraction (SPE) is applied in many protocols for the pre-concentration, clean-up and purification of different types of analytical samples as well as for the removal of toxic substances. Characteristic applications comprise enrichment of pesticides [1], determination of organic contaminants in water [2,3], and environmental samples in general [4]. Biological samples, e.g., human serum, can be purified to selectively enrich active compounds of pharmaceutical interest [5]. The SPE technology itself was introduced in the early 1970s in order to avoid or minimize the disadvantages of liquid-liquid extraction (LLE) [6]. Compared to LLE, SPE reduces the time required, especially in case of automation, while also allowing handling of small samples (50–100 µL). The term SPE was not mentioned until 1985 and since then the number of applications has continuosly increased.

## 2. Basis of Solid-Phase Extraction

For analytical purposes, SPE is usually performed using a small column or cartridge containing an appropriate packing. As an alternative, membranes loaded with distinct resins can be applied for SPE micro-extraction [7]. In a common practice the adsorbed materials are eluted from the resin with a small amount of solvent. The most commonly used material for SPE is chemically bonded silica, usually with a C_8_ or C_18_ organic group. On the other hand the most common polymeric resin in SPE is porous polystyrene [8]. Both silica- and polystyrene-based phases have distinct disadvantages: (1) silica is unstable to alkali and is known to offer only poor surface contact in case of aqueous samples due to the hydrophilic/hydrophobic balance between the carrier and the carbon chain; (2) polystyrene carriers also possess hydrophilic surfaces; (3) in most cases an activating solvent must be used, which can cause reduced capacity and/or reproducibility, respectively; (4) many types of organic compounds are incompletely extracted from aqueous solutions. Therefore, there is ongoing work with the aim of creating new types of chemically bonded resins to overcome these drawbacks.

As one of the first improvements, in 1992 Sun and Fritz modified PS-DVB with alcohol and acetyl functional groups [9]. According to the chemical character of the functional group chemically bonded to the copolymer, the resulting phases are classified as non-polar, polar, or ion exchangers. Since then a number of different stationary phases based on different types of materials—poly(carboxylic acid) coated silica [10,11], divinylbenzene-*n*-vinylpyrrolidone copolymer [12], poly(glycidyl methacrylate-divinylbenzene) (GMA-DVB) [13,14], fullerene-silica [15], titanium oxide coated steel targets [16], aluminium silicate embedded in poly(styrene-co-divinylbenzene) (PS-DVB) [17,18], zirconium silicate [19], poly(N-vinylimidazole/ethylene glycol dimethacrylate) [20]—have been designed for the selective extraction of, e.g., pharmaceutically active compounds from medicinal plants. An evaluation of commercial available SPE materials for the selective analysis of monoterpenoids was reported by Uhlschmied *et al.* [21]. For SPE of biomolecules the number of tailored stationary phases is even higher, e.g., for phosphopeptide and biomarker analysis, cellulose [22], poly(glycidyl-methacrylate/divinylbenzene) [23,24,25,26], carbon nanotubes [27], diamond layer [28,29], fullerenes [30,31,32,33], diamond-like-carbon [34,35], titanium and zirconium oxide [36], graphitic nanofibers [37], chitosan [38] and silica lanthanum oxide [39]. Nowadays the analysis after the enrichment step is mainly carried out by mass spectrometric (MS) techniques, including matrix assisted laser desorption ionization time of flight (MALDI-TOF) [40,41,42,43], electrospray ionization (ESI) and still high-performance liquid chromatography (HPLC) employing UV-absorbance, diode array detection and others [8]. The past, present and future of solid-phase extraction was reviewed by Buszewski *et al.* in 2012 [44] and Boonjob in 2014 [45].

Altogether, in 2015 using the SciFinder engine of the American Chemical Society (ACS) 67.010 hits can be found entering the term “solid phase extraction”. From those only 890 hits are correlated to infrared (IR) spectroscopic analysis, most of them describing a physicochemical characterization of the material itself [46,47,48] but only very few the analysis of the interesting analytes bound and/or enriched onto the material, respectively [49].

Compared to mass spectrometry (MS)-based techniques infrared spectroscopy in the wavenumber range between 400 and 13,000 cm^−1^ offers especially the advantages of fast, cheap, non-invasive analyses. Furthermore, several parameters can be analysed simultaneously, even in high-throughput mode [50]. The main disadvantage is the low sensitivity compared to MS in the percentage or upper ppm range. and in specific investigations the spectral selectivity for certain ingredients can be too low [51]. Therefore, the combination of SPE with IR spectroscopy could offer several synergistic benefits: the high selectivity and sensitivity of SPE could be hyphenated with the fast and simple analytical investigation using IR spectroscopy.

Therefore, the aim of the present contribution is to summarise, highlight and discuss attempts to apply this type of SPE-IR approach. In the following section the SPE setups and then NIR- and ATR-based analytical measurement technologies are summarized and discussed before in the following section different approaches divided into distinct application fields are pointed out and evaluated.

The principle of SPE is analogous to that of LLE, involving a partitioning of compounds between two phases. The analytes to be extracted are partitioned in this case between a solid and a liquid and must have a greater affinity for the solid extraction phase than for the sample matrix (retention or adsorption step). In one of the following steps compounds retained on the solid phase can be removed by eluting with a solvent with an increased affinity for the analytes. The experimental procedure normally consists of the following steps: (1) activation of the sorbent; (2) removal of the activation solvent; (3) application of the sample; (4) removal of interfering compounds; (5) selective elution of analytes. Possible uses of SPE are removal of interfering compounds; pre-concentration of samples; fractionation samples into different compound classes and storage of compounds. Thereby, the SPE procedure itself can be carried out either on-line or off-line. An important parameter for the efficiency of a SPE procedure is the breakthrough volume where the analyte of interest starts to be eluted [8].

In the classical setup, SPE can be carried out either in a column, membrane and/or micro-extraction technology, respectively. Membranes or disks consist of an e.g., 0.5 mm thick membrane, whereas the adsorbent is immobilized in a web of micro-fibrils allowing higher flow-rates compared to columns/cartridges. The most frequently used membrane worldwide is Empore from 3M (St. Paul, MN, USA) in case of large sample volumes and low analyte concentration. Solid-phase micro-extraction (SPME) has been most frequently used for the analysis of volatile and semi-volatile compounds in water [52] and is of less interest in combination with IR-related technologies so far.

## 3. Principles of Infrared Spectroscopic Analysis Techniques

IR radiation is the region of the electromagnetic spectrum between the visible (VIS) and the microwave wavelengths [53]. In near-infrared (NIR) spectroscopy (750–2500 nm, 4000–13,000 cm^−1^) solid, liquid or even gaseous samples can absorb parts of the incoming IR radiation resulting in a specific fingerprint or spectrum [54]. C–H, C–O, C=O, N–H and O–H functional groups are excited to perform stretching-, deformation- and scissor-vibrations. In contrast to mid-infrared (MIR, 4000–400 cm^−1^), where only fundamental vibrations (“signatures”) can be observed, overtones and combination vibrations are obtained in the NIR region resulting in additional information compared to MIR [55]. This mostly results in a crowded spectrum with overlapping peaks being 10–1000 times lower and less sensitive than for the MIR, which makes the construction of highly sensitive detectors necessary. The main advantage of NIR is the simultaneous determination of qualitative and quantitative parameters in a fast and non-invasive manner. Additionally, physico-chemical parameters including particle size, porosity and specific surface area can be investigated, respectively. Multivariate statistical analysis (MVA) is a powerful method enabling the selective extraction of the required information. Most appropriate chemometrical procedures include principal component analysis (PCA) and partial least square regression (PLSR). For the establishment of suitable quantitative models, reference analyses based on liquid chromatography (LC), wet chemistry, mass spectrometry (MS) and others are required. In attenuated total reflection (ATR) spectroscopy solid and liquid samples can be analyzed without any further sample pre-treatment, mainly in the MIR-region. Fourier-transform infrared (FTIR) spectroscopic microscopy can be used for the investigation of the samples’ composition with a resolution down to approximately 4 µm [56]. Finally, nowadays there is a strong trend towards the miniaturization of spectrometers [57].

### 3.1. Near-Infrared (NIR) SPECTROSCOpy vs. Mid Infrared Spectroscopy

“Near” is related on the position of the electromagnetic energy lying next to or near the visible (Vis) energy range. Molecular vibrations in the MIR cover absorptions in a range between 400–4000 cm^−1^ and are known to be the most intense and fundamental bands [51]. Light can interact with the sample in different ways. It can be reflected, scattered and absorbed or it can partly penetrate through the sample. The ratio of the portion reflected and scattered back upon interactions, *i.e.*, the remitted radiation power IR, to the light intensity incident on the sample is defined as the reflectance R:

(1) $$R=\frac{I_{R}}{I_{0}}$$

Reflectance *R* is in most applications then converted into the absorbance *A_R_*:

(2) $$A_{R}=-log_{10}\left(\frac{I_{R}}{I_{0}}\right)=-log_{10}R$$

Transmittance *T*, for measurements of light intensities transmitted through the sample is defined as:

(3) $$Τ=\frac{I_{T}}{I_{0}}=10^{-\varepsilon_{A}cd}$$

This enables one to establish the following equation:

(4) $$A=\varepsilon_{A}cd=-log_{10}\left(\frac{I_{T}}{I_{0}}\right)=-log_{10}T$$

So finally the respective absorbance can be expressed as:

(5) $$A_{T}=-log_{10}\left(\frac{1}{T}\right)\;and\;A_{R}=-log_{10}\left(\frac{1}{R}\right)$$

Beer’s law can be applied to determine the thickness of a membrane layer and the pathlength-concentration in terms of mass per unit area.

**Figure 1:**
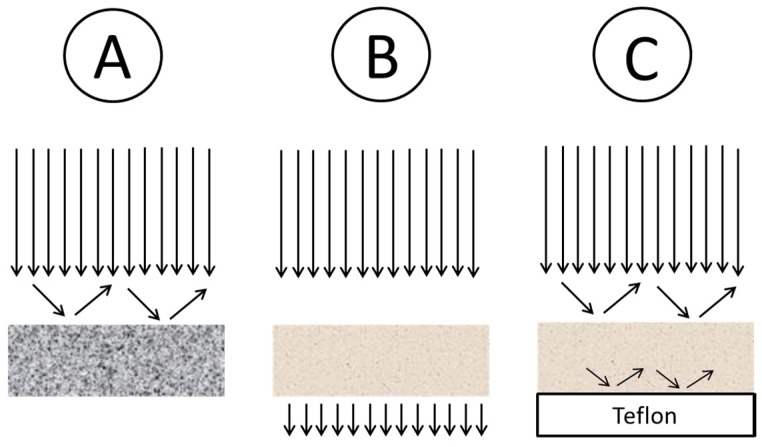
Measurement principles of (**A**) diffuse reflection, (**B**) transmission and (**C**) transflection mode.

Transflectance *T* represents a special case of transmission and reflection measurement. This measurement mode is often used for in- and on-line measurements of liquids or solutions. The incoming light beam passes the sample, then gets reflected on a non-absorbing mirror substance (in many cases Teflon) and is directed to the detector after penetrating the sample a second time. Due to this doubling of the path length, the sensitivity of the measurement setup can be enhanced (Figure 1).

The absorbance log(1/*T*) according to Beer law is rigorously only valid for non-scattering materials. In case of solid samples (powders, bulk samples) showing scattering effects, the absorbance is a so called pseudo-absorbance not exactly following the Beer law.

#### 3.1.1. Miniaturization in NIR and MIR

The development of miniaturized, handheld instruments would not only lead to a further extension of the range of applications but also suggests that these instruments could eventually appear in non-traditional user environments. When handheld mid-IR spectrometers first appeared, the military adopted them much more quickly than industry for on-site chemical quality and process control in homeland security and antiterrorism applications.

In the majority of the applications, the primary objective is rapid and safe on-site identification of unknown—often hazardous or toxic—materials or for authenticating the origin of goods by trained users—but not necessarily scientists [58].

When miniaturizing an analytical instrument one of the key objectives is to ensure that the reduction in size does not compromise measurement performance and precision. It therefore follows that handheld vibrational spectrometers will only have a real impact on quality and process control if the spectra obtained from them are comparable to those produced by larger bench-top instruments [57,59].

New developments are driven chiefly by the potential and advantages of micro-electro-mechanical systems (MEMS) production for building extremely miniaturized devices that can perform optical-mechanical functions. Handheld mid-IR instruments are limited to attenuated total reflection (ATR) measurements and are offered as FT-IR systems or with a linear variable filter (LVF) monochromator. NIR spectrometers can operate in diffuse reflection, transmission or transflection mode and their enabling technology varies between dispersive and LVF or digital light processing (DLP) using digital mirror devices (DMD).

Nevertheless, it is necessary to critically scrutinize the technical background of new, elaborately advertised products, particularly those offering investment opportunities and life-science applications, because several of them might lack feasibility by lacking selectivity and/or sensitivity, respectively.

**Figure 2:**
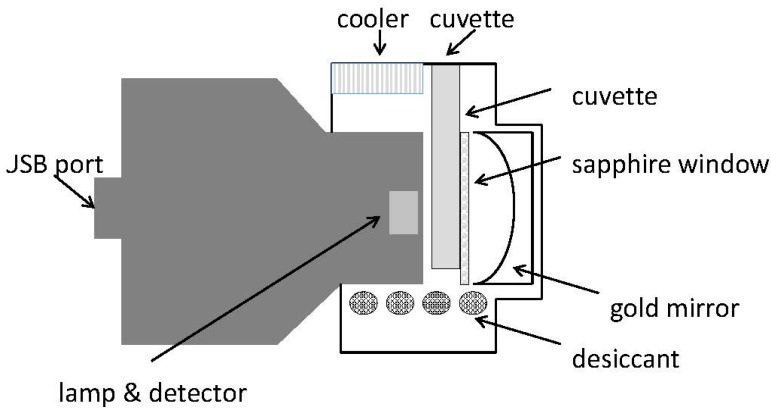
Enhanced detection system for a low-weight (<60 g) NIR probe [57].

Lutz *et al.* described one approach how to further improve the efficiency of a miniaturized light-weight (<60 g) handheld spectrometer by the modification of an InGaAs diode array detector by utilizing a thermoelectric cooling (TEC) to ensure constant measurement conditions and a gold-coated spherical mirror as a reflector superior to conventional e.g., Spectralon. With this new measurement setup ethanol in gasoline could be analyzed down to a lower limit of detection (LOD) being 0.68% whereas with the conventional detector the LOD before was 8.68% (Figure 2) [57].

#### 3.1.2. Multivariate Data Analysis in NIR and MIR

Multivariate data analysis (MVA)-based calibration procedures are carried out in order to link some spectral data with target parameters deriving from the individual reference technique [60,61,62]. In many applications, principal component analysis (PCA) models based on principal component regression (PCR) and partial least square regression (PLSR) are taken into consideration [17]. Also multiple linear regression algorithms (MLR) are applied quite frequently. In order to establish reliable calibration curves, spectra interpretation techniques including band assignment have to be combined into one single approach [63]. For the choice of the most appropriate signal, wavelength selection and spectra pretreatment algorithms are also essential [64]. Very well-known so far are the early contributions by Savitzky and Golay for data pretreatment [65]. The details about how the individual algorithms are working on a mathematical basis can be found in the most appropriate literature [60].

### 3.2. Attenuated Total Reflection (ATR) Spectroscopy

**Figure 3:**
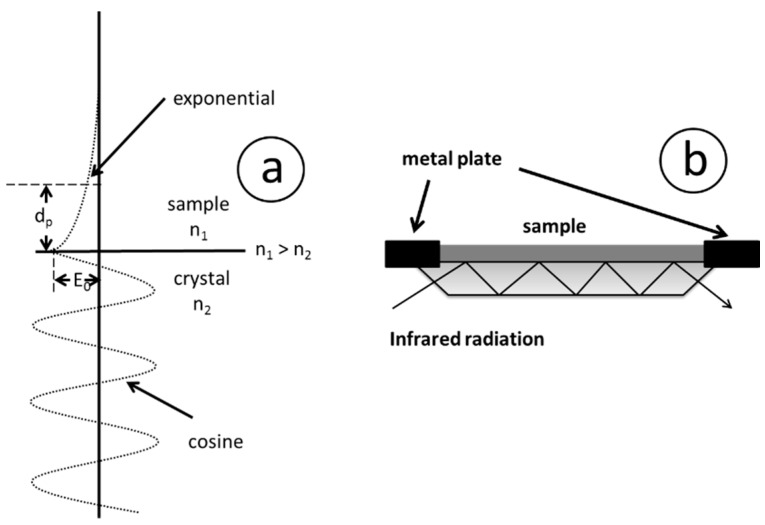
(**a**) Illustration of the evanescent wave; (**b**) principle of attenuated total reflection (ATR).

Attenuated total reflection (ATR) is mainly but not only a measurement technique applied in conjunction with mid infrared spectroscopy. ATR offers very often the advantage that solid and even liquid state samples can be directly measured without any sample pretreatment procedures necessary [66]. Applying this technique, light undergoes multiple internal reflections in a crystal of high refractive index, in many cases diamond, germanium or zinc selenite is the material of choice. The result of total internal reflection is the so called evanescent wave (Figure 3a). With this technique, infrared light is passed through the ATR crystal in such a way that it reflects at least once in most attempts even more often the internal surface in contact with the sample (Figure 3b). The depth of the evanescent wave is depending onto the exact wavelength, angle of incidence (α) and the refraction indices for the ATR crystal and the sample of interest according to the following equation [67]:

(6) dp=λn12πsin2α−(n2/n1)2

The signal-to-noise ratio (SNR) obtained depends on the number of reflections but also on the total length of the optical light path which dampens the intensity.

The advantages of ATR spectroscopy can be summarized as follows:

- Non-soluble materials can be analyzed (e.g., duroplasts)
- No KBr pellet is required
- Relatively high penetration depth for optical transparent materials
- Improved signal to noise ratio compared to measurement using KBr pellet

### 3.3. Imaging and Mapping Spectroscopy

Imaging and mapping spectroscopy has become very popular since its introduction in 1989 [68]. With the introduction of focal plane array (FPA) systems, in the beginning indium gallium arsenide (InGaAs) in 1994 [69], the subsequent development for both mid- and near-IR, using a mercury cadmium telluride (MCT), allowed the fast recording of corresponding images.

#### 3.3.1. Instrumental Setup

In principle this technique can be understood as the coupling of a microscope to an IR spectrometer. During measurement, diffraction, refraction, reflection and absorption effects play a much more predominant role than in its macroscopic counterpart. From the spectral point of view, a light source (single polychromatic thermal source), a splitter (Fourier transform (FT), tunable filter or diffraction grating), a detector (uncooled InGaAs or cooled mercury cadmium telluride (MCT)) are required [70]. The advantages of this technique at the current state of the art can be summarized as micro spatial imaging of highly complex samples, high sensitivity, high selectivity, fast data acquisition, simple sample preparation and analysis. Finally, fully automated examination and computer enhanced visualization can be accomplished [71].

#### 3.3.2. Measurement Modes

The measurement can be carried out either in the imaging or mapping mode according to the following strategies:

(1) Point mapping: different areas of the sample are analyzed consecutively
(2) Line mapping: defines a series of spectra along one dimension
(3) Area mapping: defines a series of spectra to be collected in two dimensions. Sampling of large areas requires multiple positioning of a sample

Spectra can either be recorded in transmission according to Lambert-Beer or in reflection according to Kubelka-Munk.

#### 3.3.3. Hyper Spectral Cube

**Figure 4:**
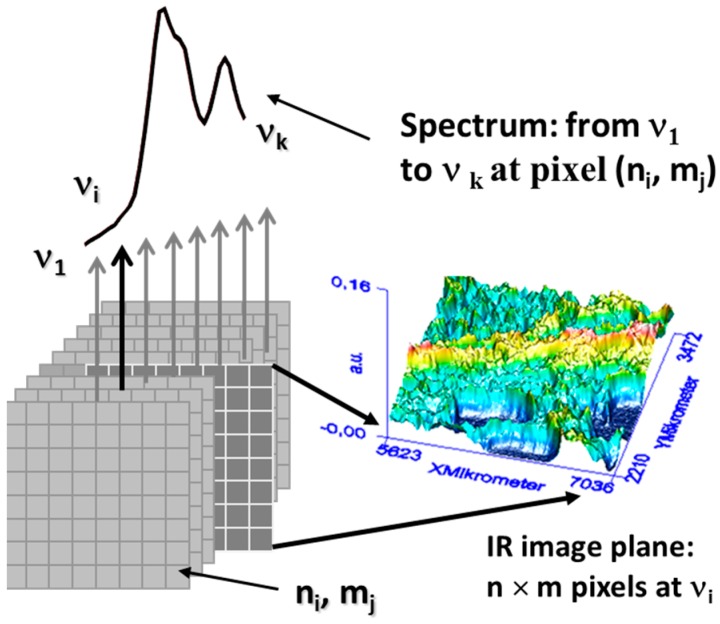
Schematic illustration of the hyperspectral cube principle.

The huge amount of data assembled in especially imaging spectroscopy is collected in a hyper spectral cube. In this cube the sample is compartmented into small surface or volume areas (referred to as pixels), each of them representing a full spectrum. These cubes are mostly displayed as a three-dimensional (3D) matrix or data cube spanning two spatial dimensions x and y. The third dimension z corresponds to the individual wavelength/wavenumber (Figure 4).

#### 3.3.4. Spectral Treatments in Imaging/Mapping Spectroscopy

At first irregularly shaped baseline contributions must be reduced, anomalous pixel spectra (outliers) and instrumental variations must be reduced. For visualization of the spatial distribution of individual ingredients, either univariate and/or multivariate analysis can be applied, respectively. For univariate analysis, a slice of the image on a particular wavenumber is displayed. In multivariate image analysis (MIA) the whole spectral information being available in the hyper spectral cube is used. Calculation of clusters are based on applying different algorithms including hierarchical clustering analysis (HC), k-means (KM) and fuzzy-C-means cluster analysis [72].

## 4. Surface-Enhanced Infrared Absorption (SEIRA) Spectroscopy

It was the field of surface-enhanced Raman scattering (SERS) that led to the establishment of a novel analytical platform termed as surface-enhanced infrared absorption (SEIRA) spectroscopy [73,74]. SEIRA is explained as being the result of the enhanced optical fields at the surface of the particles when illuminated at the surface photon resonance frequencies. In analogy with, the interpretation accepted for SERS, it has been suggested that, electromagnetic and chemical contributions are responsible for the observed infrared enhancement. It can be assumed that there is an increase in the rate of absorption per unit volume that is proportional to the energy density of the field at the appropriate density. The enhanced local field augments this energy density at the surface of particles where the adsorbed molecule resides. This local field varies according to several factors, including size, shape, and the dielectric functions, among several others. Further consequences for the observed infrared spectrum from molecules adsorbed at these local fields might be the result of enhanced field polarization. SEIRA can offer the following advantages:

- The sought sample enrichment along the solid surface reduces the content of water in the observed volume as the strong absorption of liquid water often hampers the IR spectroscopic analysis [75]
- Only those molecules that reside within the optical near-field are probed [73]
- Chip-based technology can be implemented [76]

### 4.1. Preparation of SEIRA Substrates

Enhancement of SEIRA depends on the size, shape and particle density of the selected material. In most cases a metal-underlay configuration is used. The supporting substrates can be divided into IR transparent materials (Si, Ge, CaF_2_, BaF_2_, KBr, ZnSe, ZnS, sapphire, MgO) and non-transparent materials including glass, glassy carbon, polymers, and metals. The materials of the first group enable measurement in transmission and/or reflection mode, respectively, while the others are only adequate for reflection measurements. SEIRA active metal islands are commonly prepared by high vacuum evaporation of the metal onto a supporting substrate. As an alternative, electrochemical deposition can be applied. Applying this method, the substrate on which the metal is to be deposited must have high electrical conductivity. Coinage metals Ag and Au, which show high enhancement factors in SERS, have also been the most widely used metals in SEIRA experiments. However, theoretical SEIRA models predict enhancement on transition metals being as strong as on coinage metals [77]. In fact, while Ag and Au island films lead in practice to the greatest intensity enhancement, other metals, e.g., the platinum-group metals show the modest enhancement accompanied with maximal band asymmetry.

### 4.2. Theoretical Models for SEIRA

The electromagnetic enhancement on rough metal surfaces has been extensively discussed for radiation in both the visible and near-infrared (NIR) region of the spectrum, but fewer investigations have been achieved for the mid- and far-infrared regions. Theoretical models for SEIRA include isolated, finite number of particle and surface and film models. Several achievements have been made by a number of authors and the interested reader is therefore referred to some corresponding literature [73].

### 4.3. Classical Applications of SEIRA

SEIRA is a technique similar to SERS, which is a powerful tool for structural characterization of ultrathin films and well-ordered monolayers on metal surfaces. Thin films at interfaces are prepared with different procedures and developed for various applications. The fabrication and characterization of ultrathin films is an exciting area of research in which some of the most interesting subjects are: (1) mono- and bilayers at a liquid-liquid interface; (2) adsorption monolayers at an air-water interface; (3) films and monolayers at a liquid-solid interface; (4) deposit films at an air-solid interface. In the literature one can find studies about the molecular organization of monolayers of porphyrin derivatives, of azamacrocycles, and their metallic derivatives [73].

In addition, a modest enhancement factor (10–100) makes IR spectroscopy an attractive and powerful analytical tool. The analytical application of SEIRA as a surface-sensitive spectroscopy requires a thorough examination of metal-molecule interactions and polarization effects that may give rise to a distinct vibrational spectrum, in many cases, quite different from that of the parent molecule. Peak positions and relative intensities in the enhanced spectra may be different from those of normal spectra of the same molecules, and the deviation is larger for strongly chemisorbed analytes. This means that the databases of normal IR spectra cannot be used directly for automatic identification of compounds through their SEIRA spectra and that new database must be built. The enhancement itself is short range, being most effective for molecules on or near the metal surface, and therefore, the band intensity in SEIRA is not a linear function of the amount of molecules.

In trace analysis, SEIRA has been used as a detector for flow-injection systems and applied in the analysis of environmentally hazardous chemicals in waste water [78]. Silver islands on zinc selenite as SEIRA-active surfaces have been successfully employed for coupling to liquid chromatography (LC) and gas chromatography (GC) [73]. Detection limits with then times lower in comparison with GC-FTIR have been reported for chemisorbed molecules.

Two fungicides thiram and ziram being known to be hazardous have been analyzed [79] and also several bio analytical applications including immunoassays have been described in the literature [80]. Biosensors use antibodies or enzymes, immobilized on a platform, to interact selectively with antigens or substrates, and then one of several possible transduction mechanisms to detect this interaction. Applications describing the sensitive detection of *Salmonella* and staphylococcal protein A have been described [73]. Several different applications applying SEIRA have been described in the review by Gonzalvez including a description upon the analysis down to the ppb level in different matrices such as water and soil [81].

## 5. Recent Applications Using Selective Enrichment

In the following subsections several approaches using stationary phases designed for the selective enrichment of compounds in the fields of bio-, food and environmental analysis will be described.

### 5.1. Bioanalysis

**Figure 5:**
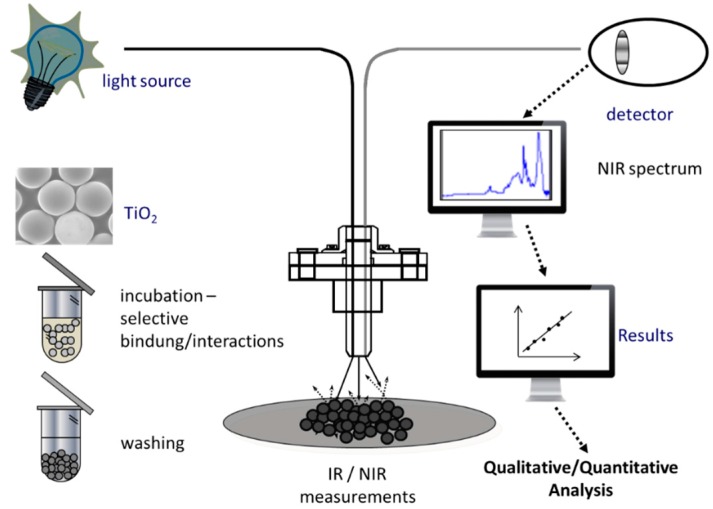
Principle of material-enhanced infrared spectroscopy (MEIRS).

In 2009 Petter *et al.* introduced a methodology termed material-enhanced infrared spectroscopy (MEIRS) for the fast quantification of low-density and high-density lipoproteins (LDL and HDL) in human serum [49]. A key risk factor in the development of atherosclerosis is a high concentration of serum LDL. Titanium oxide (TiO_2_) beads were used as an adsorbent for selectively immobilizing LDL and HDL-cholesterol and further analyzing the incubated and washed samples via NIR diffuse reflection spectroscopy (Figure 5). A principal component regression (PCR) model of 24 LDL standards in a range from 100 to 1000 ppm (clinical value is 400 ppm) was computed. The wavenumber region between 4000 cm^−1^ and 7240 cm^−1^ was found comprising the main information regarding the TiO_2_-LDL and TiO_2_-HDL composites. The regression coefficients (r) for LDL and HDL were >0.99 (calibration curve) and >0.97 (validation curve), respectively. The PCR model of TiO_2_-LDL showed a standard error of estimation (SEE) of 122.80 ppm and a standard error of prediction (SEP) of 121.15 ppm while the PLSR model of TiO_2_-HDL showed 47.70 and 47.14 ppm, respectively. In order to analyze the concentration of HDL in real serum samples, LDL was removed by adding a precipitation reagent containing 10 mg/mL magnesium dextran-sulfate, followed by incubation and centrifugation. The standard deviation (SD) for the analysis of real samples showed a value of <10%.

### 5.2. Food Analysis

Xie *et al.* reported in 2012 a method for the fast determination of trace dimethyl fumarate in milk with NIR spectroscopy following fluidized bed enrichment [82]. The motivation for the work carried out was to improve the sensitivity in NIR spectroscopy. Macroporous styrene resin HZ-816 was used as adsorbent material, and 1L solution of dimethyl fumarate was run through the material for concentration. The milk sample was pretreated to remove interfering matters such as protein, fat and then passed through the material for enrichment. After this, diffuse reflection NIR spectra were measured for the analyte concentration on the material without any elution process. The enrichment and spectral measurement procedures were easy to operate. NIR spectra in 900–1.700 nm were collected for dimethyl fumarate solutions in the concentration range of 0.506–5.060 micogram/mL and then used for multivariate calibration with partial least square (PLS) regression. Spectral pretreatment methods such as multiplicative scatter correction, first derivative, second derivative, and their combinations were carried out to select the optimal PLS model. Root mean square error of cross-validation calculated by leave-one-out cross-validation is 0.430 µg/mL. Measuring ten samples in an independent test-sample set showed a maximum deviation of 5.3%. This application clearly demonstrates how NIR coupled with on-line enrichment can be expanded for the determination of trace analytes even for applications in real liquid samples like milk.

In 2012 Chen *et al.* published an article about the determination of trace diisooctylphtalate in drink by NIR spectroscopy coupled with membrane enrichment technique [83]. Thereby, the plasticizer diisooctylphthalate (DEHP) containing solutions were passed through and concentrated with the polyethersulfone (PES) membrane and then the surface was detected directly. DEHP could be detected in a concentration range of 0.5–5.0 mg/L with a value for SEP being 0.232 mg/L.

Armenta and Lendl have introduced a flow through sensor benefiting on the selective enrichment employing silica C18 for the selective analysis of caffeine down to a level of 1.8 mg L^−1^ [84]. The principle of this work is based on a previous paper published by Orega-Barrales *et al.* in 1999 [85].

Alcudia-Leon *et al.* reported a novel ATR sensor that integrates SPE and IR detection by the design of a flow cell enabling the online coupling with a sequential injection system. As a stationary phase LiChrolut EN was located in the sensitive element of the ATR without using any external coating. This new technical attempt was evaluated for the qualitative and quantitative analysis of caffeine in soft drinks. The authors reported that the LOD was 7 µg/mL and additionally the method showed high precision, with a RSD of 4 percent. In this article not only the advantages but also the disadvantages of this novel approach have been discussed in great detail [86].

### 5.3. Environmental Analysis

Shen *et al.* reported the application of NIR spectroscopy to detect residues of the pesticide phoxim [87]. Phoxim is an organophosphate insecticide produced by the Bayer Corporation. It is allowed for use in limited applications in the European Union. It is banned for use on crops in the EU since 2007 and is applied in veterinary medicine to treat ectoparasitic acarids, which belong to the mite family. Silica gel was employed as absorbent to extract and enrich the low concentration samples, which were subsequently analyzed in diffuse reflection mode. Calibration models were established based on PLSR and leave-one-out cross validation in a range between 0.25 and 1.15 mg/mL and allowed the analysis of the organophosphate insecticide in real samples with an r for calibration being 0.924.

Regan *et al.* already reported in 1996 on the determination of pesticides in water using ATR-FTIR spectroscopy on PVC/chloroparaffin coatings [88]. The objective of the described study was the development of a novel *in situ* optical sensor consisting of PVC with a chloroparaffin plasticizer capable of enriching chlorinated pesticides and concentrating them within the penetration depth of the evanescent region of the ATR crystal of optical fiber. Measurements were performed by filling the sample cell with the aqueous pesticide solutions and recording a series of spectra over a period of time. Absorbance spectra were recorded for atrazine at 1577 cm^−1^ and alachlor at 1104 cm^−1^. Limits of detection in the region of 2 ppm have been achieved for atrazine and alachlor using a polymer-coated ATR element.

Wei *et al.* published in 2015 an article about the application of NIR spectroscopy coupled with fluidized bed enrichment and chemometrics to detect low concentration of β-naphthalenesulfonic acid in water [89]. D301 resin was used as an adsorption material to pre-concentrate in solutions in a concentration range of 2.0–100.0 μg/mL. NIR spectra were measured directly relative to the β-naphthalenesulfonic acid adsorbed on the material. An improved partial least squares (PLS) model was attained with the aid of multiplicative scatter correction pretreatment and stability competitive adaptive reweighted sampling wavenumber selection method. The root mean square error of cross validation was 1.87 μg/mL at PLS factor of 7. An independent test set was used to assess the model, with the relative error (RE) in an acceptable range of 0.46 to 10.03% and mean RE of 3.72%. This study confirmed the viability of the proposed method for the measurement of a low content of β-naphthalenesulfonic acid in water.

SPME coupled with MIR spectroscopy has been investigated for the determination of volatile organic compounds in water [90]. Standard solutions of benzene, chlorobenzene, toluene, chloroform, and *p*-chlorotoluene were prepared by spiking into methanol and diluting in water. In this study, Parafilm was used as a solid phase; it was suspended in the headspace of jars containing the aqueous solution which was then stirred. After this extraction process, FT-IR spectra of the films were obtained. Limits of detection in the range 66 ppb–1.3 ppm were reported, and reproducible extractions were achieved with a 30 min extraction time. In order to validate the procedure, the method was also applied to real water samples spiked with benzene-toluene-xylene (BETX) compounds. It was reported that solid matter within the real water samples did not significantly affect the extraction and detection of the tested compounds.

SPME has been employed in combination with FTIR for estimation of the oil and grease content in water [91]. In this study, several oils and greases were examined including *n*-hexadecane, *n*-tetradecane, *n*-nonadecane and *n*-docosane. Contaminated water samples were made by adding oil or grease samples to distilled water in a test tube, and polytetrafluoroethylene (PTFE) disks were used as a solid phase. The PTFE disks were suspended in the headspace of test tubes containing the contaminated water samples and the assembly was placed in an oven to facilitate extraction. After the extraction phase, which lasted from 1 to 14 h, transmission spectra of the PTFE disks were obtained. The authors identified CH stretching bands in the spectra of the PTFE disks after extraction, and observed that the position of the maximum corresponding to the CH stretch changed according to the contaminant studied, thus allowing identification of the different oils studied. The effect of the water matrix was studied by using three different types of water (tap, Milli-Q and seawater) and it was reported that the water matrix did not cause any significant difference in detection ability.

Silva *et al.* reported in 2008 the use of a PVC sensing phase in combination with FTIR spectroscopy for the detection of BTEX compounds in water [92]. The PVC sensing phase was placed in a vial which was filled with the aqueous solution. The studied films reached saturation after 180 min; however, 60 min enrichment was sufficient to generate a signal strong enough to measure the selected analytes. The addition of a plasticiser to the film was shown to improve the sensitivity of the method and limits of detection ranging from 4 ppm (for xylene) to 9 ppm (for ethylbenzene) were reported.

Heinrich *et al.* in 1990 were among the first to demonstrate the potential of ATR-FTIR spectroscopy combined with polymer coatings generated directly on the IRE surface for quantification of organic compounds in aqueous solution [93]. Aqueous solutions of halogenated hydrocarbons (e.g., C_2_Cl_4_, CHCl_3_) were obtained by injecting them into methanol to generate a solution. In the following distilled water was added. Both gaseous and liquid phase spectral measurements were obtained and a variety of polymer membranes were studied. Linear relationships were found between absorbance at characteristic wavenumbers and concentration of the analyte of interest. Limits of detection were generally lower for the liquid (ranging from 1 to 10 ppm) than for the gaseous (ranging from 4 to 740 ppm) phase.

Yang and Chen developed in 2001 a sensor for detecting phenolic compounds in water using SPMEs combined with FT-IR spectroscopy [94]. The SPMEs were coated directly on to ZnSe IREs and poly(acrylonitrile-*co*-butadiene) was found to be the most suitable polymer among those tested. Standard aqueous solutions of phenols were used for calibration development and detection limits of lower than 200 ppb were reported, although sensitivity was lower for high polarity compounds, such as phenol, 3-hydroxlyphenol and 2,4-dinitrophenol. This calibration technique was applied to environmental water samples and it was demonstrated that the variable water matrix in such samples did not significantly affect the detection ability. Soaking the SPME phase in water containing 5% methanol for 20 min was found to be sufficient for its regeneration.

Polyisobutylene has shown promise as a membrane for enrichment of volatile organic compounds from aqueous solutions. Yang and Tsai in 2002 used this polymer, coated on a ZnSe ATR crystal for determination of chloroform, trichloroethylene (TCE), toluene, chlorobenzene (CB) and 1-chloronaphthalene (1-CN) in aqueous solutions [95]. The solution was heated to liberate the volatile organic compounds from the sample and a cooling unit was integrated in the system to prevent warming up of the ATR crystal. Limits of detection in the ppm range were reported in this study. 

In 2004 Karlowatz *et al.* employed ethylene/propylene copolymer films coated onto Zn-Se crystals as an enriching phase for ATR-IR quantification of BTEX compounds in water. Aqueous solutions containing mixtures of BTEX compounds were passed through a flow cell in direct contact with the coated crystal. Coating the surface of the internal reflection waveguide (ZnSe crystal) with the ethylene/propylene copolymer facilitated direct detection of the compounds, which exhibited well separated absorption features in the MIR wavelength range. Limits of detection varying between 20 (xylene)–80 (toluene) ppb were reported [96].

The development of molecularly imprinted polymers (MIPs) that selectively extract specific molecules in the presence of others facilitates improved sensitivity of enrichment-based MIR methods for water quality monitoring. One of the first reported papers in this area came from Jakusch *et al.* in 1997, who developed MIPs selective for the herbicide 2,4-dichlorophenoxyacetic acid (2,4-D) [97]. The MIP film was generated on a ZnSe ATR crystal, mounted into a flow cell through which analyte solutions were pumped. Saturation of the film was achieved after 15 min and the sorption process could be completely reversed using a buffer solution. Limits of detection ranging from 3 to 210 μmoles were reported, depending on the wavenumber range employed in the analysis. 

More recently, Flavin *et al.* developed an MIR sensing methodology in which the properties of the sensing phase can be modified [98]. This is achieved using the sol-gel process in which variations of sol-gel precursors and processing conditions facilitate tailoring of the polymer properties, such as porosity, functionality and polarity. A novel phenyl-trimethoxysilane diphenyldimethoxysilane medium for detection of *p*-nitrochlorobenzene was designed. The medium was coated on a ZnSe ATR crystal which was placed in a temperature controlled flow cell. Analyte solutions were pumped over the polymer surface. The residence time required for this method was reduced by increasing the temperature of the system, and limits of detection of 0.7 ppm were reported.

Subsequent developments in the use of silver halide fibres for water quality monitoring have been reported. Gobel *et al.* employed tapered silver halide FOs to improve the sensitivity of FEWS measurement of chlorinated hydrocarbons in water. Decreasing the fibre diameter increases evanescent water absorbtion and thus the sensitivity of the system. The tapered fibres were coated in polyisobutylene (PIB) and TeCE was used as a test analyte in this study. A minimum limit of detection of 50 ppb was achieved using the tapered fibres, while that for non-tapered fibres was in the range 100–300 ppb. The time taken for regeneration of the sensor in this case was around 10 min [99].

Current methods for pesticide detection require sample enrichment followed by GC/GC-MS/LC/enzyme immunoassay and are unsuitable for field testing, continuous monitoring or screening. As a consequence, there has been much interest in the development of MIR sensors for pesticide detection in water. Regan *et al.* developed a sensor that could detect pesticides in water employing a polymer film coated on either an ATR crystal or silver halide FO coated in a polymer film. Atrazine and alachlor were used as test analytes in this study; the polymer film (PVC with a chloroparaffin pasticiser) enriched their concentration by up to a factor of 30. With the proposed system, detection limits of 2 ppm were achievable [88]. 

Mizaikoff in 1999 reported the development of an MIR-FEWS sensing system for pollution monitoring in the subsea environment [100]. This consisted of an FTIR spectrometer coupled to a silver halide optical fiber sensor coated with E/PCo. A conventional lab-based ATR spectrometer with an E/PCo layer was used to demonstrate the feasibility of the proposed system and it was shown that it could detect chlorinated hydrocarbons in artificial seawater at low ppb (100–115 ppb) concentrations. The development of a sensor head for this system, consisting of U-shaped silver halide fibres coated with polymer membranes, has also been reported [101]. Simultaneous detection of tetrachloroethylene (TeCE), 1,2-dichlorobenzene and the three xylene isomers at concentrations ranging from 100 to 5 ppm were reported. 

Solid-phase extraction (SPE) using synthetic materials in combination with NIRS has been demonstrated for detection of a variety of organic compounds in water. Albuquerque *et al.* in 2004 measured BTEX compounds in water using a silicone rod attached to an NIR transflectance probe [102]. The probe assembly was placed in an aqueous solution containing BTEX compounds, which was mechanically stirred during measurements. Limits of detection of 8, 7, 2.6 and 3 ppm were reported for benzene, toluene, ethylbenzene and *m*-xylene, respectively. The sensor was used on gasoline and diesel fuel contaminated water samples and it was possible to discriminate between the contaminant sources using first derivative spectra.

In another study Lima *et al.* pre-extracted by means of a solid PDMS disk placed in an aqueous solution containing BTEX compounds. After agitation of the solution, the disk was removed and its NIR spectra obtained. Limits of detection of 0.080, 0.12, 0.14 and 0.27 ppm were reported for benzene, toluene, ethylbenzene and xylenes, respectively [103].

Lendl *et al.* reported in 2009 about the implementation of a new flow-through FT-IR sensor for oil in water analysis based on SPE employing silica C28 particles as a stationary phase. For this on-line approach no chlorinated solvents are required, reducing the environmental impact. Additionally, this method benefited from the minimal sample preparation steps. Spectra were recorded by co-adding 32 scans ata a resolution of 4 cm^−1^. The vibrational band located at 1462 cm^−1^ due to the CH(2) bending was integrated from 1475 to 1450 cm^−1^ and following baseline correction was established between 1485 and 1440 cm^−1^ using the areas as analytical signal. It was reported that the technique provides a limit of detection (LOD) of 22 mg L^−1^ and a precision expressed as relative standard deviation (RSD) lower than 5%. The major advantages of this novel approach are the speed and its high level of automation [104]. 

Roy *et al.* introduced in 2008 a completely different strategy for determining subnanomolar iron concentrations in ocean seawater. The introduced device uses an iron-specific chelating biomolecule, desferrioxamine B (DFB), covalently immobilized on a mesoporous silica film. Changes in infrared spectral signatures of the immobilized DFB upon Fe(III) complexation provided an reproducible and exact measure of iron on the surface of a chip that has been exposed to seawater. The LOD found was 50 pM for a 1 L sample at pH 1.7 and was used to measure dissolved iron in subarctic Pacific waters without any interference from other elements. This analytical technique provides an autonomous research platform being helpful to better understand the iron distribution and chemistry in seawater [105].

ClearSampler^TM^ (Richland, WA, USA) offers a wide range of kits consisting of a swab or scoop to pick up the sample and a collection vial with buffer to solubilize or suspend the sample [106]. Several different kits are available for analysing proteins, DNA, but also hazardous materials including anthrax, bacteria (e.g., Salmonella, *etc.*). In ASTM D7575-11 a standard test method for solvent-free membrane recoverable oil and grease by IR determination is described following this measurement principle [107].

## 6. Conclusions

Detection limits reported in the majority of vibrational spectroscopy applications have been in the ppm range, while the maximum allowable concentration of some contaminants, especially in environmental waters, is in the ppb range. This wide gap between the ppm and ppb concentration presents a major limitation to the practical widespread adoption of vibrational spectroscopic methods. At the ppm detection level, the main use of these systems would be as screening tools for wide scale monitoring. It should also be considered that these detection limits are usually quoted for experiments carried out in idealised laboratory conditions. The transfer of such sensors from the lab to real quality monitoring of real bodies is still a substantial challenge and extensive validation with real samples is required.

## Abbreviations

ATR	Attenuated total reflection
BTEX	Benzene, toluene and xylene
CB	Chlorobenzene
DEHP	Diisooctylphthalate
DLC	Diamond like carbon
DLP	Digital light processing
DMD	Digital mirror devices
ESI	Electrospray ionisation
ESI	Electrospray ionisation
FPA	Focal plane array
FT-IR	Fourier-transfrom infrared
GC	Gas chromatography
GMA-DVB	Poly(glycidyl methacrylate-*co*-divinylbenzene)
HC	Hierarchical cluster
HDL	High density lipoprotein
HPLC	High-performance liquid chromatography
KM	K-means
LDL	Low density lipoprotein
LLE	Liquid-liquid extraction
LOD	Limit of detection
LVF	Linear variable filter
MALDI	Matrix assisted laser desorption ionisation
MCT	Mercury cadmium telluride
MEMS	Micro-electro-mechanical system
MIA	Multivariate imaging analysis
MIP	Molecularly imprinted polymers
MLR	Multiple linear regression
MS	Mass spectrometry
MVA	Multivariate analysis
PCA	Principal component analysis
PDMS	Poly(dimethylsiloxane)
PES	Polyethersulfone
PIB	Polyisobutylene
PLSR	Partial least square regression
PS-DVB	Poly(styrene-*co*-divinylbenzene)
PTFE	Polytetrafluoroethylene
PVC	Poly(vinylchloride)
RE	Relative error
SD	Standard deviation
SEE	Standard error of estimation
SEIRA	Surface enhanced infrared analysis
SEP	Standard error of prediction
SERS	Surface enhanced Raman spectroscopy
SNR	Signal to noise ratio
SPE	Solid-phase extraction
SPME	Solid-phase micro extraction
TEC	Thermoelectric cooling
Vis	Visible
